# Supplementary material for: Clarifying the role of an unavailable distractor in human multiattribute choice
Source: eLife. 2022 Dec 6;11:e83316. doi: 10.7554/eLife.83316 (PMC9757826; doi:10.7554/eLife.83316)
Supplement: Supplementary file 1. [file elife-83316-supp1.docx]

**Supplemental Table 1** (related to Fig. 3). Optimal parameter estimates of dynamic models: Mean (SE) and cross-validated log-likelihood (CV LL).

**AU FFI linear (n = 6 free parameters)**

| $\lambda$ | $k$ | $\theta$ | $I_{0}$ | $t_{\mathrm{nd}}$ | $c$ | CV LL |
| --- | --- | --- | --- | --- | --- | --- |
| .475 (.017) | 3.89 (.11) | 3.21 (.097) | 2.81 (.06) | .141 (.012) | .76 (.011) | -958.5 |

**EV FFI linear (n = 5)**

| $k$ | $\theta$ | $I_{0}$ | $t_{\mathrm{nd}}$ | $c$ | CV LL |
| --- | --- | --- | --- | --- | --- |
| 3.38 (.097) | 3.076 (.09) | 3.14 (.061) | .152 (.012) | .85 (.01) | -1191.3 |

**EV (+ DN) FFI linear (n = 5)**

| $k$ | $\theta$ | $I_{0}$ | $t_{\mathrm{nd}}$ | $c$ | CV LL |
| --- | --- | --- | --- | --- | --- |
| 1.96 (.061) | 2.92 (.097) | 2.59 (.07) | .167 (.012) | .38 (.009) | -1286.1 |

**Dual-route EV linear (n = 6)**

| $k$ | $k^{DN}$ | $\theta$ | $I_{0}$ | $t_{\mathrm{nd}}$ | $f_{MI}$ | CV LL |
| --- | --- | --- | --- | --- | --- | --- |
| 5.62 (.21) | 2.53 (.11) | 2.77 (.08) | 1.99 (.064) | .169 (.011) | .75 (.021) | -1150 |

**AU FFI non-linear (n = 9)**

| $\lambda$ | $k$ | $\theta$ | $I_{0}$ | $t_{\mathrm{nd}}$ | $c$ | $\eta$ | $P_{0}$ | $\gamma$ | CV LL |
| --- | --- | --- | --- | --- | --- | --- | --- | --- | --- |
| .337 (.023) | 11.7 (.61) | 3.26 (.099) | 1.29 (.17) | .144 (.012) | .77 (.011) | 5.6 (1.04) | .69 (.032) | 4.8 (.88) | -822 |

**EV FFI non-linear (n = 8)**

| $k$ | $\theta$ | $I_{0}$ | $t_{\mathrm{nd}}$ | $c$ | $\eta$ | $P_{0}$ | $\gamma$ | CV LL |
| --- | --- | --- | --- | --- | --- | --- | --- | --- |
| 6.25 (.41) | 3.22 (.09) | 2.48 (.16) | .15 (.012) | .81 (.012) | 2.39 (.57) | .56 (.032) | 1.04 (.14) | -897 |

**EV (+ DN) FFI non-linear (n = 8)**

| $k$ | $\theta$ | $I_{0}$ | $t_{\mathrm{nd}}$ | $c$ | $\eta$ | $P_{0}$ | $\gamma$ | CV LL |
| --- | --- | --- | --- | --- | --- | --- | --- | --- |
| 3.52 (.44) | 3.07 (.096) | 2.67 (.083) | .16 (.012) | .45 (.015) | 17.98 (1.7) | .61 (.03) | 13.8 (1.4) | -1052 |

**Dual-route EV non-linear (n = 9)**

| $k$ | $k^{DN}$ | $\theta$ | $I_{0}$ | $t_{\mathrm{nd}}$ | $f_{MI}$ | $\eta$ | $P_{0}$ | $\gamma$ | CV LL |
| --- | --- | --- | --- | --- | --- | --- | --- | --- | --- |
| 6.58 (.38) | 4.29 (.40) | 2.95 (.09) | 1.69 (.12) | .16 (.011) | .67 (.027) | 9.23 (1.28) | .56 (.03) | 5.74 (.96) | -927 |
