# Supplementary material for: Clarifying the role of an unavailable distractor in human multiattribute choice
Source: eLife. 2022 Dec 6;11:e83316. doi: 10.7554/eLife.83316 (PMC9757826; doi:10.7554/eLife.83316)
Supplement: Supplementary file 2. [file elife-83316-supp2.docx]

**Supplemental Table 2** (related to Figs. 2, 3). Optimal parameter estimates of static models: Mean (SE), and cross-validated log-likelihood (CV LL).

**AU softmax linear (n = 2 free parameters)**

| $\lambda$ | $\beta$ | CV LL |
| --- | --- | --- |
| .457 (.017) | 9.15 (.302) | -2330.1 |

**EV softmax linear (n = 1)**

| $\beta$ | CV LL |
| --- | --- |
| 6.79 (.198) | -2645.5 |

**EV (+ DN) softmax linear (n = 1)**

| $\beta$ | CV LL |
| --- | --- |
| 3.35 (.11) | -2656.4 |

**AU softmax non-linear (n = 5)**

| $\lambda$ | $\beta$ | $\eta$ | $P_{0}$ | $\gamma$ | CV LL |
| --- | --- | --- | --- | --- | --- |
| .327 (.019) | 16.4 (.47) | 4.72 (.938) | .669 (.031) | 4.43 (.792) | -2193.3 |

**EV softmax non-linear (n = 4)**

| $\beta$ | $\eta$ | $P_{0}$ | $\gamma$ | CV LL |
| --- | --- | --- | --- | --- |
| 33.9 (8.81) | 3.69 (.74) | .524 (.036) | .82 (.13) | -2388.8 |

**EV (+ DN) softmax non-linear (n = 4)**

| $\beta$ | $\eta$ | $P_{0}$ | $\gamma$ | CV LL |
| --- | --- | --- | --- | --- |
| 31.3 (6.53) | 13.5 (1.62) | .607 (.031) | 9.44 (1.24) | -2481.2 |
