# Supplementary material for: Clarifying the role of an unavailable distractor in human multiattribute choice
Source: eLife. 2022 Dec 6;11:e83316. doi: 10.7554/eLife.83316 (PMC9757826; doi:10.7554/eLife.83316)
Supplement: Supplementary file 3. — *Significant effects (p < 0.05; two-sided one-sample t-tests of GLM coefficients against 0) following Holm’s sequential Bonferroni correction for multiple comparisons. C: ‘Condition’ (binary vs. ternary). [file elife-83316-supp3.docx]

**Supplemental Table 3** (related to Fig. 2). GLMs using subjective additive-utility (AU) regressors. * Significant effects (*p* < .05; 2-sided 1-sample *t*-tests of GLM coefficients against 0) following Holm’s sequential Bonferroni correction for multiple comparisons. C: ‘Condition’ (binary vs. ternary).

*AU*_D_ *as (absolute) distractor variable (with ‘AU sum’ covariate):*

|  | T value | p-value (uncorrected) |
| --- | --- | --- |
| *AU*_H_ – *AU*_L_ | 12.3 | < .0001* |
| *AU*_H_ + *AU*_L_ | 11.9 | < .0001* |
| *AU*_D_ | 2.37 | .0193 |
| (*AU*_H_ – *AU*_L_) x *AU*_D_ | -.099 | .92 |
| (*AU*_H_ – *AU*_L_) x C | -6.09 | < .0001* |
| (*AU*_H_ + *AU*_L_) x C | -5.24 | < .0001* |
| *AU*_D_ x C | -2.27 | .024 |
| (*AU*_H_ – *AU*_L_) x *AU*_D_ x C | -1.64 | .102 |
| C | -5.04 | < .0001* |

*AU*_D_ *as (absolute) distractor variable (without ‘AU sum’ covariate):*

|  | T value | p-value (uncorrected) |
| --- | --- | --- |
| *AU*_H_ – *AU*_L_ | 13.9 | < .0001* |
| *AU*_D_ | 3.93 | < .001* |
| (*AU*_H_ – *AU*_L_) x *AU*_D_ | -.44 | .66 |
| (*AU*_H_ – *AU*_L_) x C | -6.56 | < .0001* |
| *AU*_D_ x C | -2.87 | .0047* |
| (*AU*_H_ – *AU*_L_) x *AU*_D_ x C | -1.74 | .083 |
| C | -8.06 | < .0001* |

*AU*_D_ *– AU*_H_ *as (relative) distractor variable (with ‘AU sum’ covariate):*

|  | T value | p-value (uncorrected) |
| --- | --- | --- |
| *AU*_H_ – *AU*_L_ | 13.1 | < .0001* |
| *AU*_H_ + *AU*_L_ | 11.7 | < .0001* |
| *AU*_D_ *– AU*_H_ | -.35 | .73 |
| (*AU*_H_ – *AU*_L_) x (*AU*_D_ *– AU*_H_) | -4.25 | < .0001* |
| (*AU*_H_ – *AU*_L_) x C | -6.24 | < .0001* |
| (*AU*_H_ + *AU*_L_) x C | -5.63 | < .0001* |
| (*AU*_D_ *– AU*_H_) x C | -1.86 | .065 |
| (*AU*_H_ – *AU*_L_) x (*AU*_D_ *– AU*_H_) x C | -.54 | .59 |
| C | -5.39 | < .0001* |

*AU*_D_ *– AU*_H_ *as (relative) distractor variable (without ‘AU sum’ covariate):*

|  | T value | p-value (uncorrected) |
| --- | --- | --- |
| *AU*_H_ – *AU*_L_ | 13.7 | < .0001* |
| *AU*_D_ *– AU*_H_ | -10.2 | < .0001* |
| (*AU*_H_ – *AU*_L_) x (*AU*_D_ *– AU*_H_) | -5.65 | < .0001* |
| (*AU*_H_ – *AU*_L_) x C | -6.41 | < .0001* |
| (*AU*_D_ *– AU*_H_) x C | 1.95 | .054 |
| (*AU*_H_ – *AU*_L_) x (*AU*_D_ *– AU*_H_) x C | .22 | .83 |
| C | -7.18 | < .0001* |
