# Supplementary material for: Clarifying the role of an unavailable distractor in human multiattribute choice
Source: eLife. 2022 Dec 6;11:e83316. doi: 10.7554/eLife.83316 (PMC9757826; doi:10.7554/eLife.83316)
Supplement: Supplementary file 4. [file elife-83316-supp4.docx]

**Supplemental Table 4** (related to Fig. 4). Optimal parameter estimates of context-dependent dynamic models: Mean (SE), and cross-validated log-likelihood (CV LL).

**SI AU FFI (n = 8 free parameters) [Ternary]**

| $\lambda$ | SI w_2_ | SI w_3_ | $k$ | $\theta$ | $I_{0}$ | $t_{\mathrm{nd}}$ | $c$ | CV LL |
| --- | --- | --- | --- | --- | --- | --- | --- | --- |
| .534 (.019) | .25 (.029) | .43 (.034) | 2.44 (.078) | 4.73 (.108) | 3.82 (.074) | .024 (.006) | .786 (.02) | -1310.5 |

**SI AU FFI (n = 7) [Binary]**

| $\lambda$ | SI w_2_ | $k$ | $\theta$ | $I_{0}$ | $t_{\mathrm{nd}}$ | $c$ | CV LL |
| --- | --- | --- | --- | --- | --- | --- | --- |
| .487 (.016) | .31 (.02) | 3.54 (.11) | 3.21 (.097) | 2.91 (.061) | .145 (.012) | .74 (.015) | -915 |

**Adaptive Gain AU FFI (n = 10) [Ternary]**

| $\lambda$ | $b_{p}$ | $s_{p}$ | $b_{x}$ | $s_{x}$ | $k$ | $\theta$ | $I_{0}$ | $t_{\mathrm{nd}}$ | $c$ | CV LL |
| --- | --- | --- | --- | --- | --- | --- | --- | --- | --- | --- |
| .43 (.027) | -.24 (.12) | .23 (.04) | .033 (.12) | .24 (.056) | 44.2 (5.84) | 4.7 (.11) | .54 (.44) | .023 (.006) | .56 (.034) | -1530 |

**Adaptive Gain AU FFI (n = 10) [Binary]**

| $\lambda$ | $b_{p}$ | $s_{p}$ | $b_{x}$ | $s_{x}$ | $k$ | $\theta$ | $I_{0}$ | $t_{\mathrm{nd}}$ | $c$ | CV LL |
| --- | --- | --- | --- | --- | --- | --- | --- | --- | --- | --- |
| .42 (.023) | -.16 (.039) | .15 (.02) | -.092 (.074) | .18 (.051) | 16.3 (2.4) | 3.16 (.09) | -2.68 (.43) | .15 (.012) | .19 (.02) | -1012 |

**Dual-route EV (n = 6) [Ternary]**

| $k$ | $k^{DN}$ | $\theta$ | $I_{0}$ | $t_{\mathrm{nd}}$ | $f_{MI}$ | CV LL |
| --- | --- | --- | --- | --- | --- | --- |
| 3.21 (.15) | 1.68 (.09) | 4.16 (.097) | 2.85 (.065) | .035 (.008) | .72 (.028) | -1579 |

**Dual-route EV (n = 6) [Binary]**

| $k$ | $k^{DN}$ | $\theta$ | $I_{0}$ | $t_{\mathrm{nd}}$ | $f_{MI}$ | CV LL |
| --- | --- | --- | --- | --- | --- | --- |
| 5.62 (.21) | 2.53 (.11) | 2.77 (.08) | 1.99 (.064) | .169 (.011) | .75 (.021) | -1150 |
